# The Impact of Binge Drinking on Mortality and Liver Disease in the Swiss HIV Cohort Study

**DOI:** 10.3390/jcm10020295

**Published:** 2021-01-14

**Authors:** Bernard Surial, Nicolas Bertholet, Jean-Bernard Daeppen, Katharine E. A. Darling, Alexandra Calmy, Huldrych F. Günthard, Marcel Stöckle, Enos Bernasconi, Patrick Schmid, Andri Rauch, Hansjakob Furrer, Gilles Wandeler, The Swiss HIV Cohort Study

**Affiliations:** 1 Department of Infectious Diseases, Inselspital, Bern University Hospital, University of Bern, 3010 Bern, Switzerland; andri.rauch@insel.ch (A.R.); hansjakob.furrer@insel.ch (H.F.); gilles.wandeler@insel.ch (G.W.); 2 Addiction Medicine, Department of Psychiatry, Lausanne University Hospital, University of Lausanne, 1011 Lausanne, Switzerland; nicolas.bertholet@chuv.ch (N.B.); jean-bernard.daeppen@chuv.ch (J.-B.D.); 3 Division of Infectious Diseases, University Hospital of Lausanne, University of Lausanne, 1011 Lausanne, Switzerland; katharine.darling@chuv.ch; 4 Division of Infectious Diseases, Geneva University Hospital, University of Geneva, 1205 Geneva, Switzerland; alexandra.calmy@hcuge.ch; 5 Division of Infectious Diseases and Hospital Epidemiology, University Hospital Zurich, University of Zurich, 8091 Zurich, Switzerland; huldrych.guenthard@usz.ch; 6 Institute of Medical Virology, University of Zurich, 8057 Zurich, Switzerland; 7 Division of Infectious Diseases and Hospital Epidemiology, University Hospital Basel, University of Basel, 4031 Basel, Switzerland; marcel.stoeckle@usb.ch; 8 Division of Infectious Diseases, Regional Hospital of Lugano, 6903 Lugano, Switzerland; enos.bernasconi@eoc.ch; 9 Division of Infectious Diseases, Cantonal Hospital of St. Gallen, 9007 St. Gallen, Switzerland; patrick.schmid@kssg.ch; 10 Institute of Social and Preventive Medicine, University of Bern, 3012 Bern, Switzerland

**Keywords:** alcohol, binge drinking, HIV, liver-related outcomes, mortality

## Abstract

Whereas excessive alcohol consumption increases liver disease incidence and mortality, evidence on the risk associated with specific drinking patterns is emerging. We assessed the impact of binge drinking on mortality and liver disease in the Swiss HIV Cohort Study. All participants with follow-up between 2013 and 2020 were categorized into one of four drinking pattern groups: “abstinence”, “non-hazardous drinking”, “hazardous but not binge drinking” (Alcohol Use Disorder Identification Test Consumption [AUDIT-C] score ≥ 3 in women and ≥4 in men), and “binge drinking” (≥6 drinks/occasion more than monthly). We estimated adjusted incidence rate ratios (aIRR) for all-cause mortality, liver-related mortality and liver-related events using multivariable quasi-Poisson regression. Among 11,849 individuals (median follow-up 6.8 years), 470 died (incidence rate 7.1/1000 person-years, 95% confidence interval [CI] 6.5–7.8), 37 experienced a liver-related death (0.6/1000, 0.4–0.8), and 239 liver-related events occurred (3.7/1000, 3.2–4.2). Compared to individuals with non-hazardous drinking, those reporting binge drinking were more likely to die (all-cause mortality: aIRR 1.9, 95% CI 1.3–2.7; liver-related mortality: 3.6, 0.9–13.9) and to experience a liver-related event (3.8, 2.4–5.8). We observed no difference in outcomes between participants reporting non-hazardous and hazardous without binge drinking. These findings highlight the importance of assessing drinking patterns in clinical routine.

## 1. Introduction

The harmful use of alcohol has been identified as one of the leading threats for global health. The World Health Organization (WHO) estimated that in 2016, 5% of all deaths were due to the harmful use of alcohol globally, and up to one in ten deaths were attributable to the consumption of alcohol in Switzerland [1,2]. After viral hepatitis, alcohol consumption remains one of the main causes of end-stage liver disease, and accounts for 30–40% of all deaths due to liver cirrhosis in high-income countries [3]. Among people living with HIV (PLWH), the prevalence of alcohol use disorder was estimated to reach 30%, with significant differences across regions and demographic groups [4]. Excessive alcohol consumption in PLWH is an important driver of sexual risk behavior and increases the risk of virological failure to antiretroviral therapy (ART) and mortality [5,6,7]. In addition, compared to individuals without HIV, PLWH might experience increased mortality at lower levels of alcohol use [8].

Many studies have shown the association between heavy alcohol consumption and liver-related events and mortality [9,10,11]. However, it is only recently that research from large cohort studies pointed towards the impact of specific drinking patterns, especially binge drinking, on mortality and the development of advanced liver disease. In a population-based study including more than 6000 participants in Finland, researchers observed that individuals with binge drinking behavior had a substantially increased risk of developing liver disease, independent of their daily average alcohol intake [12]. Considering the high proportion of individuals reporting high alcohol consumption in cohorts of PLWH across the globe, binge drinking may well have an impact on long-term clinical outcomes in this population [13,14,15].

We took advantage of the systematic assessment of alcohol consumption patterns in a nationwide prospective cohort of PLWH to assess the impact of drinking patterns on all-cause and liver-related mortality, as well as on the occurrence of liver-related events.

## 2. Methods

### 2.1. Swiss HIV Cohort Study and Inclusion Criteria

The Swiss HIV Cohort Study (SHCS, www.shcs.ch) is an ongoing nationally representative cohort study which includes close to 80% of all PLWH receiving ART in Switzerland [16]. Participants are prospectively followed at one of five university hospitals, an affiliated hospital or by a private physician associated with the SHCS. Detailed information about sociodemographic characteristics, clinical events, risk behavior, laboratory values, and medication are recorded at study registration, and every 6 months thereafter. The SHCS was approved by the local ethical committees of the participating centers: Ethikkommission beider Basel (“Die Ethikkommission beider Basel hat die Dokumente zur Studie zustimmend zur Kenntnis genommen und genehmigt.”); Kantonale Ethikkommission Bern (21/88); Comité départemental d’éthique des spécialités médicales et de médecine communautaire et de premier recours, Hôpitaux Universitaires de Genève (01–142); Commission cantonale d’éthique de la recherche sur l’être humain, Canton de Vaud (131/01); Comitato etico cantonale, Repubblica e Cantone Ticino (CE 813); Ethikkommission des Kantons St. Gallen (EKSG 12/003); Kantonale Ethikkommission Zürich (KEK-ZH-NR: EK-793), and written informed consent was obtained from all participants.

All individuals with follow-up visits between the introduction of the routine assessment of alcohol consumption using the Alcohol Use Disorder Identification Test Consumption questionnaire (AUDIT-C, 1 January 2013) and database closure (30 April 2020) were included. Individuals without any follow-up information after the first AUDIT-C assessment were excluded.

### 2.2. Outcomes

Our primary outcome was all-cause mortality, and our secondary outcomes were liver-related mortality and the occurrence of liver-related events. In the SHCS, all deaths and clinical events are reported using standardized case-report forms and causes of death are recorded using ICD-10 codes, which are validated using medical reports. Liver-related deaths included death from hepatitis (viral, alcohol or toxic), cirrhosis, hepatocellular carcinoma (HCC), acute liver failure and variceal bleeding. Liver-related events that are routinely reported by cohort physicians are the histologic diagnosis of cirrhosis, diagnosis of portal hypertension, HCC, variceal bleeding, hepatic encephalopathy stage III or IV, hepatorenal syndrome, liver transplant, spontaneous bacterial peritonitis, and ascites confirmed on imaging. In this study, suspected liver cirrhosis based on an AST-to-platelet ratio index (APRI) above 2 on at least two consecutive cohort visits was also considered to be a liver-related event, and the date of the second cohort visit was the event date [17]. Since liver-related events are conditional on each other (e.g., diagnosis of portal hypertension is conditional on the presence of liver cirrhosis), only the first liver-related event during the follow-up period was considered for each individual.

### 2.3. Exposure

The main exposure of interest for all analyses was the self-reported alcohol consumption pattern, which was assessed using the AUDIT-C questionnaire, a simple clinical tool developed to screen for alcohol misuse [18]. It is based on three questions aimed at quantifying alcohol drinking frequency, alcohol quantity consumed on a typical occasion, and at detecting binge drinking behavior (consumption of ≥6 drinks per occasion, Table S1). A score of ≥4 for men and ≥3 for women was considered as elevated except when all points resulted from the question on drinking frequency. Based on AUDIT-C assessments, individuals were categorized into four mutually exclusive alcohol drinking pattern groups: “abstinence” if they reported to have never consumed alcoholic drinks in the preceding 6 months, “non-hazardous drinking” if they reported drinking alcohol but without an elevated AUDIT-C score, “hazardous drinking” if they had an elevated AUDIT-C score but reported to consume ≥6 drinks per occasion monthly or less, and “binge drinking” if individuals reported to consume ≥6 drinks more than monthly. Time-varying alcohol drinking patterns were used for all analyses.

### 2.4. Statistical Analyses

Patient characteristics stratified by the most frequent drinking pattern reported during follow-up were compared using Chi-squared and Kruskal–Wallis tests. Time from the first AUDIT-C assessment to the event of interest were presented using Kaplan–Meier curves. To evaluate the impact of alcohol drinking patterns on all-cause and liver-related mortality as well as on the occurrence of liver-related events, adjusted incidence rate ratios (IRR) were estimated using quasi-Poisson regression with robust standard errors. Non-hazardous drinking was used as reference since it was the most frequent drinking pattern. Multivariable analyses for all-cause mortality were adjusted for time-fixed values of age, sex, education level (no, basic, and high education), ethnicity (Caucasian vs. other), HIV transmission group (heterosexual, men who have sex with men, persons who inject drugs (PWID), and others), hepatitis B virus (HBV) infection (positive HBV surface antigen), and history of an AIDS-defining disease. Time-varying covariates included hepatitis C virus (HCV) infection (detectable HCV viral load), smoking (yes/no), any illicit drug use, presence of depression (assessed by the cohort physician), employment status (employed, housework, or unemployed), and whether individuals lived with a partner or not. Analyses on liver-related mortality were adjusted for age, sex, and HBV or HCV coinfection, and analyses on liver-related events included the same covariates as the analyses on overall mortality, with the addition of time-varying values of body mass index (BMI).

### 2.5. Sensitivity Analyses

Since PWID might be at higher risk of all-cause mortality, we repeated the analysis on all-cause mortality after excluding PWID. Since time-varying exposures insufficiently capture the cumulative impact of alcohol drinking patterns on liver-related events, we repeated the analysis using the most common drinking pattern reported within each individual’s follow-up as time-fixed exposure. In addition, we explored the robustness of our results in an analysis excluding individuals with HBV or HCV coinfection. Finally, due to the heterogeneity of our study population, we performed subgroup analyses stratified by HIV transmission group, and a formal test of interaction was performed. All analyses were performed using R version 4.0.2 [19].

## 3. Results

### 3.1. Study Population

Of 11,886 participants with follow-up after 1 January 2013, 11,849 with available follow-up data after the first AUDIT-C assessments were included. Overall, 3264 (27.5%) were female, median age was 46 years (interquartile range [IQR] 38 to 53), 9097 (76.8%) were Caucasian, and 5425 (45.8%) were men who have sex with men (MSM). Individuals provided a median of 12 AUDIT-C assessments (IQR 7 to 13). Non-hazardous drinking was the most frequent alcohol consumption pattern in 6861 individuals (57.9%), followed by abstinence in 2718 individuals (22.9%), hazardous but no binge drinking in 1814 individuals (15.3%), and binge drinking in 456 individuals (3.8%). Patient characteristics stratified by the most frequent drinking pattern reported during follow-up are shown in Table 1. Compared to individuals with non-hazardous drinking as the most frequent pattern, patients with binge drinking were more likely to be Caucasian men, PWID, to report no or basic education, to be smokers, and to be unemployed, whereas they were less likely to live with a partner, and reported higher rates of depressive episodes during follow-up. Individuals who most frequently reported abstinence were more likely to be women, had a lower nadir CD4 cell count, and were more likely to have a history of AIDS-defining disease and to have HBV or HCV coinfection than those who reported non-hazardous drinking as the most frequent pattern (*p* < 0.001 for all comparisons).

### 3.2. Alcohol Drinking Patterns

**Figure 1:**
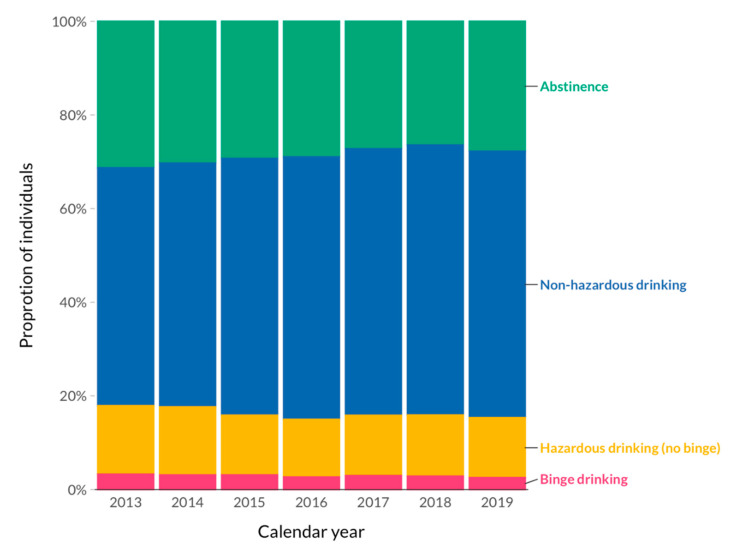
Time trends of alcohol drinking patterns over the studied period. The plot shows the proportion of alcohol drinking patterns at each calendar year of the study (excluding 2020 due to incomplete follow-up). In individuals who had more than one follow-up visit per calendar year, the most common category was recorded.

**Figure 2:**
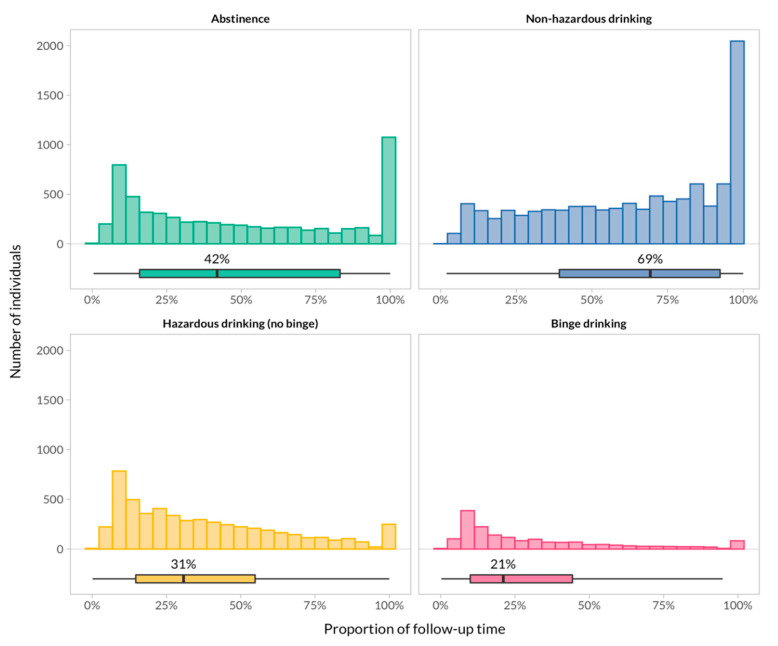
Distribution of the proportion of follow-up time spent in each category at the individual level. Histograms and boxplots show the distribution and summary of participants’ contribution to follow-up time in each category. Individuals who reported hazardous or binge drinking remained in this category for a median of 31% and 21% of their follow-up, respectively. A substantial proportion of individuals (large bars on the right) who contributed to the abstaining or non-hazardous drinking category stayed in that same category for 100% of their follow-up.

Overall, 54.6% of the follow-up time was spent in the non-hazardous drinking category, followed by 24.2% in the abstinence category, 16.8% in the hazardous drinking category, and 4.4% in the binge-drinking category. The proportion of individuals who reported hazardous and binge drinking remained stable over time (Figure 1). The individual contribution of participants to follow-up time in each drinking pattern category is shown in Figure 2. In summary, participants who contributed data to the non-hazardous drinking category stayed in this category during the majority of their follow-up time, whereas hazardous and binge-drinking patterns accounted only for a minor proportion of follow-up time in those who contributed data to these categories. A bimodal distribution was apparent for individuals who reported abstinence: A substantial proportion of individuals consistently reported abstinence for 100% of their follow-up time, and a second group of patients for less than 20% of their follow-up time.

### 3.3. All-Cause and Liver-Related Deaths

**Figure 3:**
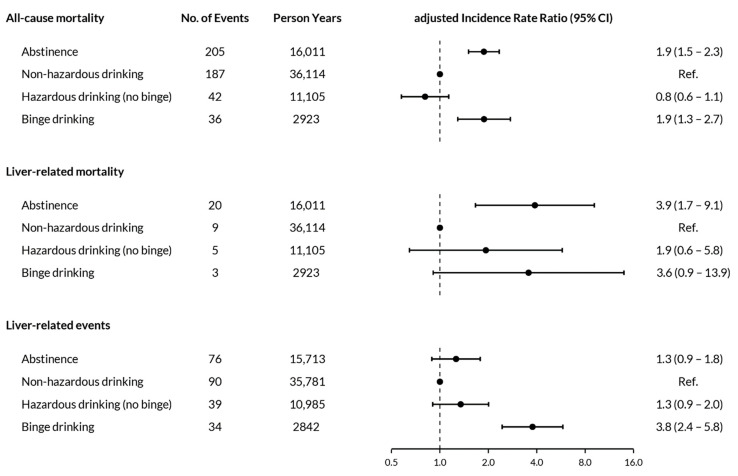
Impact of alcohol drinking pattern on all-cause mortality, liver-related mortality and liver-related events. Analyses on all-cause mortality were adjusted for age, sex, education level, ethnicity, HIV transmission group, infection with hepatitis B or C, history of AIDS-defining disease, smoking, any illicit drug use, presence of depression, employment status, and whether individuals lived together with a partner or not. Covariates for liver-related mortality included age, sex, and viral hepatitis coinfection, and analyses for liver events were adjusted for the same covariates as the analysis on overall mortality, with the addition of time-varying values of body mass index. Liver-related events included cirrhosis based on confirmed APRI >2 or histology, diagnosis of portal hypertension, hepatocellular carcinoma, variceal bleeding, hepatic encephalopathy stage III or IV, hepatorenal syndrome, liver transplant, spontaneous bacterial peritonitis and ascites confirmed on imaging.

During 66,153 person-years (PY) of follow-up, 470 individuals died (incidence rate 7.1 per 1000 person-years, 95% confidence interval [CI] 6.5 to 7.8); the most common causes of death were non-AIDS cancer (*n* = 105, 22.3%), cardiovascular (*n* = 52, 11.1%), HIV-related (*n* = 25, 5.3%), accident (*n* = 22, 4.7%), and other infections (*n* = 18, 3.8%). Liver-related deaths occurred in 37 individuals (incidence rate 0.6 per 1000 person-years, 95% CI 0.4 to 0.8). Detailed causes of liver-related deaths, stratified by drinking pattern, are shown in Table S2. All-cause deaths stratified by calendar year, HIV transmission group, and alcohol drinking pattern are shown in Table 2. All-cause mortality rates were highest among PWID (18.5 per 1000 person-years, 95% CI 15.6 to 21.9), and were higher in individuals who abstained from alcohol (12.8 per 1000 person-years, 95% CI 11.2 to 14.7) or reported binge drinking (12.3 per 1000 person-years, 95% CI 8.9 to 17.0) compared to those in other alcohol consumption categories (Figure S1). In multivariable analyses, all-cause mortality was higher among individuals reporting binge drinking (IRR 1.9, 95% CI 1.3 to 2.7) and abstinence (IRR 1.9, 95% CI 1.5 to 2.3) compared to those reporting non-hazardous drinking. Similarly, liver-related mortality was higher among PLWH reporting binge drinking (IRR 3.6, 95% CI 0.9 to 13.9) and abstinence (IRR 3.9, 95% CI 1.7 to 9.1) compared to those reporting non-hazardous drinking (Figure 3, Figure S2, Table S3). There was no evidence for a difference in all-cause mortality (IRR 0.8, 95% CI 0.6 to 1.1) or liver-related mortality (IRR 1.9, 95% CI 0.6 to 5.8) between individuals reporting hazardous (no binge) drinking and non-hazardous drinking.

### 3.4. Liver-Related Events

Within the study period, 239 individuals experienced a liver-related event (incidence rate 3.7 per 1000 person-years, 95% CI 3.2 to 4.2), with a diagnosis of cirrhosis based on a confirmed APRI score above 2 in 140 individuals (Table 3). Incidence rates were highest among individuals who reported binge drinking (12.0 per 1000 person-years, 95% CI 8.5 to 16.8), followed by abstinence (4.8 per 1000 person-years, 95% CI 3.9 to 6.1), hazardous drinking (3.6 per 1000 person-years, 95% CI 2.6 to 4.9), and non-hazardous drinking (2.5 per 1000 person-years, 95% CI 2.0 to 3.1, Table 2, Figure S3). In multivariable analyses, PLWH reporting binge drinking were more likely to experience a liver-related event (adjusted IRR 3.7, 95% CI 2.4 to 5.8) compared with those reporting non-hazardous drinking, whereas comparisons with individuals reporting abstinence (adjusted IRR 1.3, 95% CI 0.9 to 1.8) and with those reporting hazardous drinking (adjusted IRR 1.3, 95% CI 0.9 to 2.0) were not statistically significant (Figure 3).

### 3.5. Sensitivity Analyses

After excluding individuals who acquired HIV through injection drug use (*n* = 1347), all-cause mortality was higher in individuals reporting abstinence (IRR 2.1, 95% CI 1.7 to 2.7) and binge drinking (IRR 1.9, 95% CI 1.2 to 3.0) compared to those reporting non-hazardous drinking. Repeating the analyses with each individual’s most frequently reported alcohol drinking pattern over the follow-up period as time-fixed exposure, binge drinking remained strongly associated with all-cause mortality (adjusted IRR 1.8, 95% CI 1.2 to 2.7) and with the development of a liver-related event (adjusted IRR 2.8, 95% CI 1.7 to 4.8) when compared to non-hazardous drinking (Tables S3 and S4). In an analysis excluding 1649 individuals with HBV or HCV coinfection, the risk for liver-related events was more pronounced in PLWH reporting binge drinking (adjusted IRR 7.1, 95% CI 4.0 to 12.6), with a higher point estimate than in the main analysis (Table S4). In subgroup analyses stratified by HIV transmission groups, the impact of binge drinking on incidence of liver-related events was largest among heterosexual individuals and MSM but was less pronounced in PWID (*p*-value for interaction = 0.44, Figure S4).

## 4. Discussion

In this nationwide cohort study of PLWH, binge drinking was associated with an increased risk for all-cause and liver-related mortality and led to a substantially increased rate of liver-related events. Compared to individuals reporting non-hazardous drinking, those who reported abstinence had a higher risk of all-cause and liver-related mortality, confirming previous observations among PLWH and the general population.

Between 2013 and 2020, 14.8% of cohort participants reported binge drinking, which is lower than the prevalence estimated by WHO for the general population worldwide (18.2%) and in Switzerland (35.6%) [1]. These differences were probably driven by the definitions used for binge drinking: in contrast to the WHO, but in analogy with previous studies, we considered individuals who reported binge drinking more than monthly for this category in order to capture two clinically distinct alcohol drinking phenotypes [12]. In our study, binge drinking, but not hazardous drinking, was associated with a higher rate of liver-related events. This is in line with a recent study from the general population in Finland, which showed a similar association, after controlling for individual average amount of alcohol intake [12]. Failing to account for specific alcohol consumption patterns among individuals with hazardous drinking may partly explain why previous studies among PLWH were unable to establish a relationship between hazardous alcohol consumption and liver disease [20,21,22]. Whereas two studies found an increased risk for liver disease among individuals with hazardous alcohol use [21,22], associations did not remain significant after taking HCV coinfection into account. We found a strong association between binge drinking and all-cause mortality, extending findings from studies in the general population, which showed an increase in mortality due to cancer and liver disease in this subpopulation [10,11]. The marginal association between binge drinking and liver-related mortality needs to be interpreted with caution as a very limited number of liver-related deaths was observed during the study period. Altogether, our findings highlight the importance of explicitly capturing specific patterns of alcohol consumption in addition to the overall amount consumed, especially among populations with a high prevalence of co-existing liver disease.

Individuals who reported abstinence from alcohol consumption had a higher risk of all-cause and liver-related mortality than those with non-hazardous drinking. Such a “J-shaped” association has been described previously among the general population and in PLWH [7,11,23]. Classification of people with high risk of liver disease into the abstinence category might have happened in two ways: First, former drinkers, who differ from lifetime abstainers with respect to previous liver injury, might have been classified as abstainers. The increased risk of liver-related mortality among individuals reporting abstinence in our study might point towards this hypothesis. Second, individuals with serious illnesses or pre-existing liver disease might have been more likely to abstain from alcohol. In our study, individuals who abstained from alcohol were more likely to have HBV and HCV coinfection and to have had a history of AIDS-defining diseases, indicating the presence of a so-called “sick quitter” effect.

The stringent ascertainment of causes of death and the detailed availability of information about risk behavior (such as smoking) and education level (a determinant of socioeconomic status) allowed us to study the long-term impact of alcohol drinking patterns on mortality, and to adjust these analyses for important confounders. Due to the systematic and complete longitudinal assessment of alcohol drinking patterns using AUDIT-C, we were able to establish a clear relationship between binge drinking and mortality as well as the occurrence of liver-related events among PLWH. These results were robust across a range of sensitivity analyses, and after taking viral hepatitis and mode of HIV transmission into account. Some limitations, inherent to routine data collection in cohort studies, may have influenced our results: although the occurrence of predefined liver-related events is routinely reported in the SHCS, no systematic assessment of liver fibrosis/cirrhosis has been performed among individuals without viral hepatitis. Our use of APRI, a non-invasive marker for liver fibrosis, as a diagnostic marker of liver cirrhosis, may have led to the under-estimation of the rate of this outcome due to its low sensitivity [24]. Conversely, since aspartate aminotransferase levels (part of the APRI score) might be increased due to alcohol consumption per se, the number of liver-related events among hazardous and binge drinkers could have been over-estimated. Furthermore, our main exposure being self-reported, the misclassification of alcohol drinking patterns might have influenced our results. Thus, under-reporting of problematic alcohol consumption could partly explain the increased risk of all outcomes among individuals classified as abstainers.

In conclusion, our study suggests higher mortality rates and an increased risk of liver-related events associated with binge drinking among PLWH, a population with a high prevalence of alcohol use disorder and pre-existing liver disease. Our findings confirm the results of studies in the general population and warrant the inclusion of detailed alcohol pattern assessments into clinical routine to identify individuals at highest risk for complications. Our results could help tailor risk reduction counseling based on alcohol drinking patterns to the specific needs of PLWH. Studies with longitudinal assessment of liver fibrosis among PLWH are needed to fully grasp the impact of alcohol drinking patterns on the development of liver cirrhosis in this population.

## Figures and Tables

**Table 1:** Description of the study population, stratified by the most frequently reported alcohol drinking pattern.

	Abstinence	Non-Hazardous Drinking	Hazardous Drinking(No Binge)	Binge Drinking
*n*	2718	6861	1814	456
Female (%)	1220 (44.9)	1697 (24.7)	287 (15.8)	60 (13.2)
Median age, years (IQR)	47 (39–53)	47 (39–53)	45 (36–52)	46 (36–52)
Caucasian (%)	1756 (64.6)	5436 (79.2)	1529 (84.3)	376 (82.5)
HIV transmission (%)				
Heterosexual	1333 (49.0)	2455 (35.8)	502 (27.7)	145 (31.8)
PWID	516 (19.0)	559 (8.1)	197 (10.9)	75 (16.4)
MSM	692 (25.5)	3483 (50.8)	1037 (57.2)	213 (46.7)
Other	177 (6.5)	364 (5.3)	78 (4.3)	23 (5.0)
Education (%)				
No education	332 (12.2)	333 (4.9)	85 (4.7)	35 (7.7)
Basic education	1658 (61.0)	3838 (55.9)	976 (53.8)	266 (58.3)
Higher education	604 (22.2)	2528 (36.8)	722 (39.8)	145 (31.8)
*Missing data*	124 (4.6)	162 (2.4)	31 (1.7)	10 (2.2)
Median nadir CD4, cells/µL (IQR)	186 (79–294)	222 (110–338)	241 (126–380)	226 (117–373)
History of AIDS defining disease (%)	744 (27.4)	1430 (20.8)	305 (16.8)	96 (21.1)
HBV coinfection (%)	165 (6.1)	327 (4.8)	66 (3.6)	15 (3.3)
HCV coinfection (%)	372 (13.7)	525 (7.7)	158 (8.7)	61 (13.4)
Smoker (%)	1203 (44.3)	2975 (43.4)	1097 (60.5)	336 (73.7)
Unemployed (%)	1311 (48.2)	1757 (25.6)	450 (24.8)	201 (44.1)
Lives with partner (%)	1139 (41.9)	3351 (48.8)	782 (43.1)	158 (34.6)
Any depressive episode (%)	1186 (43.6)	2263 (33.0)	649 (35.8)	216 (47.4)
BMI category (%)				
Normal	1377 (50.7)	3840 (56.0)	1075 (59.3)	260 (57.0)
Underweight	205 (7.5)	274 (4.0)	77 (4.2)	27 (5.9)
Overweight/obese	1046 (38.5)	2624 (38.2)	633 (34.9)	158 (34.6)
*Missing data*	90 (3.3)	123 (1.8)	29 (1.6)	11 (2.4)
Median follow-up, years (IQR)	6.8 (3.8–7.0)	6.8 (4.6–7.1)	6.7 (3.3–7.0)	6.2 (3.1–7.0)

For each individual, the most commonly recorded alcohol drinking pattern, unemployment status, and partnership status during follow-up are shown in this table. Individuals who reported to have ever smoked during the follow-up were considered to be smokers. Age refers to the age at first AUDIT-C assessment. IQR: interquartile range; PWID: persons who inject drugs; MSM: men having sex with men; HBV: hepatitis B virus; HCV: hepatitis C virus; BMI: body mass index.

**Table 2:** Incidence rates of deaths and liver-related events.

	All-Cause Deaths			Liver-Related Deaths		Liver-Related Events		
Variable	Person-Years	*n*	Rate per 1000 Person-Years (95% CI)	*n*	Rate per 1000 Person-Years (95% CI)	Person-Years	*n*	Rate per 1000 Person-Years (95% CI)
Overall	66,153	470	7.1 (6.5–7.8)	37	0.6 (0.4–0.8)	65,321	239	3.7 (3.2–4.2)
Calendar year ^1^								
2013	8482	65	7.7 (6.0– 9.8)	7	0.8 (0.4–1.7)	8459	70	8.3 (6.5–10.5)
2014	9252	60	6.5 (5.0–8.4)	8	0.9 (0.4–1.7)	9176	44	4.8 (3.6–6.4)
2015	9526	96	10.1 (8.3–12.3)	5	0.5 (0.2–1.3)	9417	37	3.9 (2.8–5.4)
2016	9647	69	7.2 (5.7–9.1)	6	0.6 (0.3–1.4)	9510	30	3.2 (2.2–4.5)
2017	9603	83	8.6 (7.0–10.7)	6	0.6 (0.3–1.4)	9451	28	3.0 (2.0–4.3)
2018	9660	66	6.8 (5.4–8.7)	4	0.4 (0.2–1.1)	9496	23	2.4 (1.6–3.6)
2019	9352	30	3.2 (2.2–4.6)	1	0.1 (0.0–0.8)	9194	7	0.8 (0.4–1.6)
2020	592	1	1.7 (0.2–12.0)	0	0	578	0	0
Transmission risk group								
Heterosexual	25,289	145	5.7 (4.9–6.7)	11	0.4 (0.2–0.8)	25,089	63	2.5 (4.9–6.7)
PWID	7466	138	18.5 (15.6–21.9)	21	2.8 (1.8–4.3)	7098	100	14.1 (15.6–21.9)
MSM	30,072	165	5.5 (4.7–6.4)	5	0.2 (0.1–0.4)	29,839	68	2.3 (4.7–6.4)
Other	3326	22	6.6 (4.4–10.0)	0	0	3294	8	2.4 (4.4–10.0)
Alcohol drinking pattern								
Abstinence	16,011	205	12.8 (11.2–14.7)	20	1.2 (0.8–1.9)	15,713	76	4.8 (3.9–6.1)
Non-hazardous drinking	36,114	187	5.2 (4.5–6.0)	9	0.2 (0.1–0.5)	35,781	90	2.5 (2.0–3.1)
Hazardous drinking (no binge)	11,105	42	3.8 (2.8–5.1)	5	0.5 (0.2–1.1)	10,985	39	3.6 (2.6–4.9)
Binge drinking	2923	36	12.3 (8.9–17.0)	3	1.0 (0.3–3.2)	2842	34	12.0 (8.5–16.8)

PWID: persons who inject drugs, MSM: men who have sex with men, CI: confidence interval; ^1^ Database closure was in April 2020.

**Table 3:** Liver-related events.

Liver-Related Event	*n*
Cirrhosis (confirmed AST-to-platelet ratio index (APRI-score) above 2)	140
Cirrhosis (based on liver-biopsy)	28
Diagnosis of portal hypertension	20
Hepatocellular carcinoma (HCC)	20
Bleeding from gastric or esophageal varices	10
Hepatic encephalopathy (stage III or IV)	9
Hepatorenal syndrome	4
Liver transplantation	3
Spontaneous bacterial peritonitis (with cirrhosis)	3
Ascites, clinically and confirmed by imaging	2
**Total**	239

## Data Availability

According to the Swiss law, data cannot be shared if data subjects have not agreed or data is too sensitive to share. Investigators with a request for selected data should send a proposal to the respective SHCS address (www.shcs.ch/contact). The provision of data will be considered by the Scientific Board of the SHCS and the study team and is subject to Swiss legal and ethical regulations, and is outlined in a material and data transfer agreement.

## Supplementary Materials

The following are available online at https://www.mdpi.com/2077-0383/10/2/295/s1, Table S1: Alcohol Use Disorders Identification Test Consumption Score (AUDIT-C), Table S2: Detailed causes of death of individuals who experienced a liver-related death. Table S3: Incidence rate ratios (IRR) of all-cause and liver-related deaths, by alcohol drinking pattern, Table S4: Incidence rate ratios (IRR) for liver-related events, by alcohol drinking pattern, Figure S1. Kaplan–Meier curves showing the time from the first AUDIT-C assessment to all-cause death, stratified by alcohol drinking pattern. Figure S2. Kaplan–Meier curves showing the time from the first AUDIT-C assessment to liver-related death, stratified by alcohol drinking pattern. Figure S3. Kaplan–Meier curves showing the time from the first AUDIT-C assessment to the first liver-related event, stratified by alcohol drinking pattern. Figure S4: Subgroup analyses of the incidence rate ratios (IRR) for the occurrence of liver-related events by HIV transmission group.
