# Supplementary material for: The Impact of Binge Drinking on Mortality and Liver Disease in the Swiss HIV Cohort Study
Source: J Clin Med. 2021 Jan 14;10(2):295. doi: 10.3390/jcm10020295 (PMC7830571; doi:10.3390/jcm10020295)
Supplement: Supplementary file 1 [file jcm-10-00295-s001.pdf]

Supplementary Material

Table S1. Alcohol Use Disorders Identification Test Consumption Score (AUDIT-C)

Item	Answer	Score
1) How often did you consume alcoholic drinks?	Never	0
	Monthly or less	1
	2-4 times per month	2
	2-3 times per week	3
	≥4 times per week	4
2) How many drinks[1] did you consume on a typical occasion?	1 or 2 drinks	0
	3–4 drinks	1
	5–6 drinks	2
	7–9 drinks	3
	10 drinks or more	4
3) How frequently did you drink 6 or more alcohol drinks?	Never	0
	Less than monthly	1
	Monthly	2
	Weekly	3
	(Almost) daily	4

The AUDIT-C score is a clinical screening tool to detect alcohol misuse. A total score of ≥3 for women and ≥4 for men is considered as potentially hazardous drinking.

[1]Alcoholic drinks refer to standard drinks, which contain 10-12 grams of ethanol, e.g. 1.5 dL of wine or 3.3 dL of beer.

Table S2. Detailed cause of death of individuals who experienced a liver-related death

Cause of Death	Abstaining	Non-hazardous drinking	Hazardous drinking (no binge)	Binge drinking
**Acute liver failure**	2 (11.1%)	1 (8.3%)	1 (20.0%)	2 (100.0%)
**Hepatocellular carcinoma**	9 (50.0%)	6 (50.0%)	2 (40.0%)	0
**Hepatorenal syndrome**	2 (11.1%)	0	0	0
**Liver cirrhosis**	2 (11.1%)	3 (25.0%)	2 (40.0%)	0
**Variceal bleeding**	1 (5.6%)	0	0	0
**Viral hepatitis**	2 (11.1%)	2 (16.7%)	0	0
**Total**	18 (100.0%)	12 (100.0%)	5 (100.0%)	2 (100.0%)

Table S3. Incidence rate ratios (IRR) of all-cause and liver-related deaths, by alcohol drinking pattern

Alcohol drinking pattern	All cause death			Liver-related death	
	Univariable IRR (95% CI)	Time-varying model[1] IRR (95% CI)[1]	Time-fixed model[2] IRR (95% CI)	Univariable IRR (95% CI)	Multivariable IRR (95% CI)[3]
**Abstinence**	2.5 (2.0 to 3.0)	1.9 (1.5 to 2.3)	1.4 (1.1 to 1.8)	5.0 (2.3 to 11.0)	3.9 (1.7 to 9.1)
**Non-hazardous drinking**	Ref.	Ref.	Ref.	Ref.	Ref.
**Hazardous drinking (no binge)**	0.7 (0.5 to 1.0)	0.8 (0.6 to 1.1)	1.4 (1.0 to 1.8)	1.8 (0.6 to 5.4)	1.9 (0.6 to 5.8)
**Binge drinking**	2.4 (1.7 to 3.4)	1.9 (1.3 to 2.7)	1.8 (1.2 to 2.7)	4.1 (1.1 to 15.2)	3.6 (0.9 to 13.9)

[1]The main exposure was the time-varying alcohol drinking pattern, and the analysis was adjusted for age, sex, ethnicity, HIV transmission group, nadir CD4 cell count, education, HBV and HCV co-infection, history of Aids defining disease, depressive episodes, employment status, partnership, smoking and illicit drug use. [2]The time-fixed model used each individual's most common alcohol drinking pattern as exposure and used the same covariates as the time-varying model. [3]Adjusted for age, sex, HBV and HCV coinfection. **CI** confidence interval, **HBV** hepatitis B virus, **HCV** hepatitis C virus. **CI** confidence interval, **HBV** hepatitis B virus, **HCV** hepatitis C virus, **CDC** center for disease control.

Table S4. Incidence rate ratios (IRR) for liver-related events, by alcohol drinking pattern

Alcohol drinking pattern	Univariable IRR (95% CI)	Time-varying model[1] IRR (95% CI)	Time-fixed model[1] IRR (95% CI)	Without HBV/HCV[1] IRR (95% CI)
**Abstinence**	1.9 (1.4 to 2.6)	1.3 (0.9 to 1.8)	1.2 (0.8 to 1.7)	1.4 (0.8 to 2.5)
**Non-hazardous drinking**	Ref.	Ref.	Ref.	Ref.
**Hazardous drinking (no binge)**	1.4 (1.0 to 2.1)	1.3 (0.9 to 2.0)	2.4 (1.7 to 3.4)	1.7 (0.9 to 2.9)
**Binge drinking**	4.8 (3.2 to 7.1)	3.8 (2.4 to 5.8)	2.8 (1.7 to 4.8)	7.1 (4.0 to 12.6)

[1]Adjusted for age, sex, ethnicity, transmission group, nadir CD4 cell count, education, HBV and HCV co-infection, history of Aids defining disease, BMI, smoking, depression, employment status, partnership and illicit drug use. **CI** confidence interval, **HBV** hepatitis B virus, **HCV** hepatitis C virus, **BMI** body mass index.

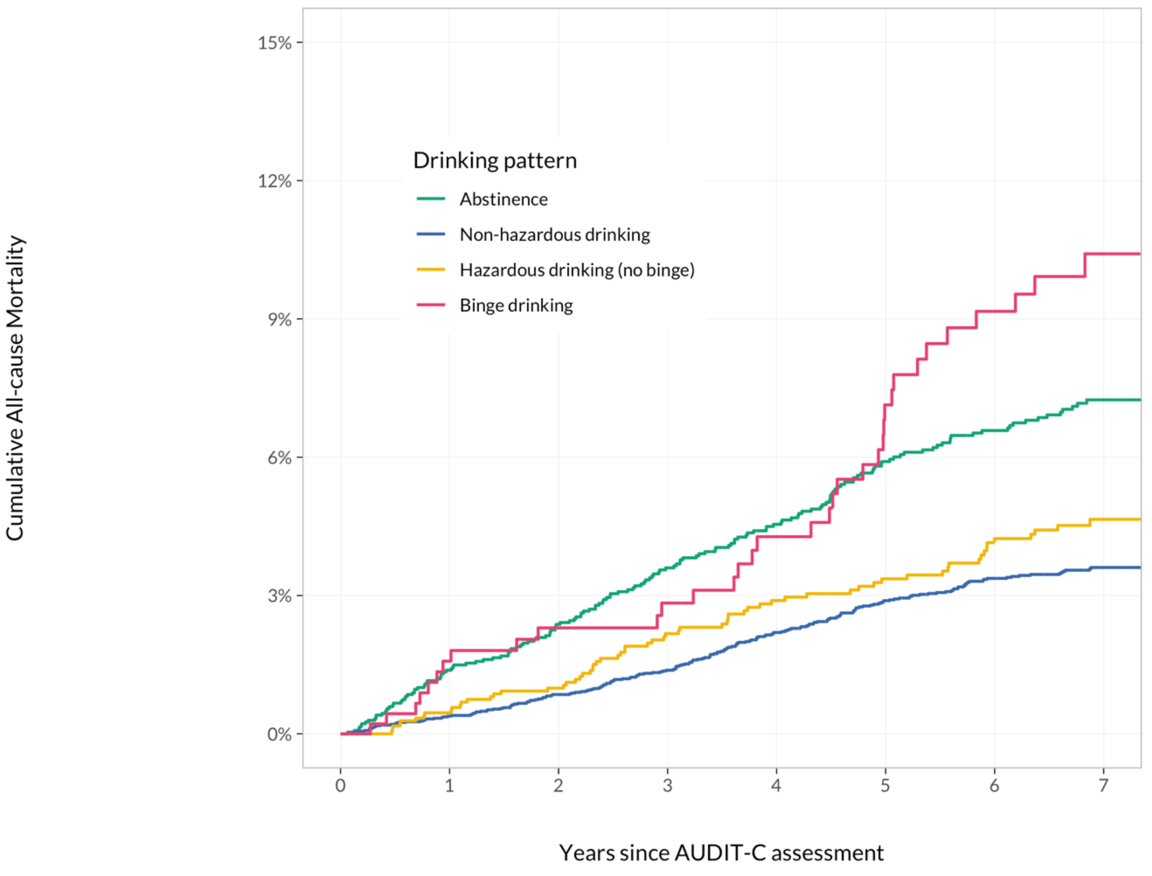

Individuals at risk

	0	1	2	3	4	5	6	7
Abstinence	2723	2589	2389	2214	2043	1883	1712	766
Non-hazardous drinking	6865	6590	6185	5818	5437	5029	4612	2194
Hazardous drinking (no binge)	1801	1714	1564	1432	1311	1190	1072	473
Binge drinking	460	427	388	361	319	286	251	112

Years since AUDIT-C assessment

Figure S1. Kaplan Meier curves showing the time from the first AUDIT-C assessment to all-cause death, stratified by alcohol drinking pattern.

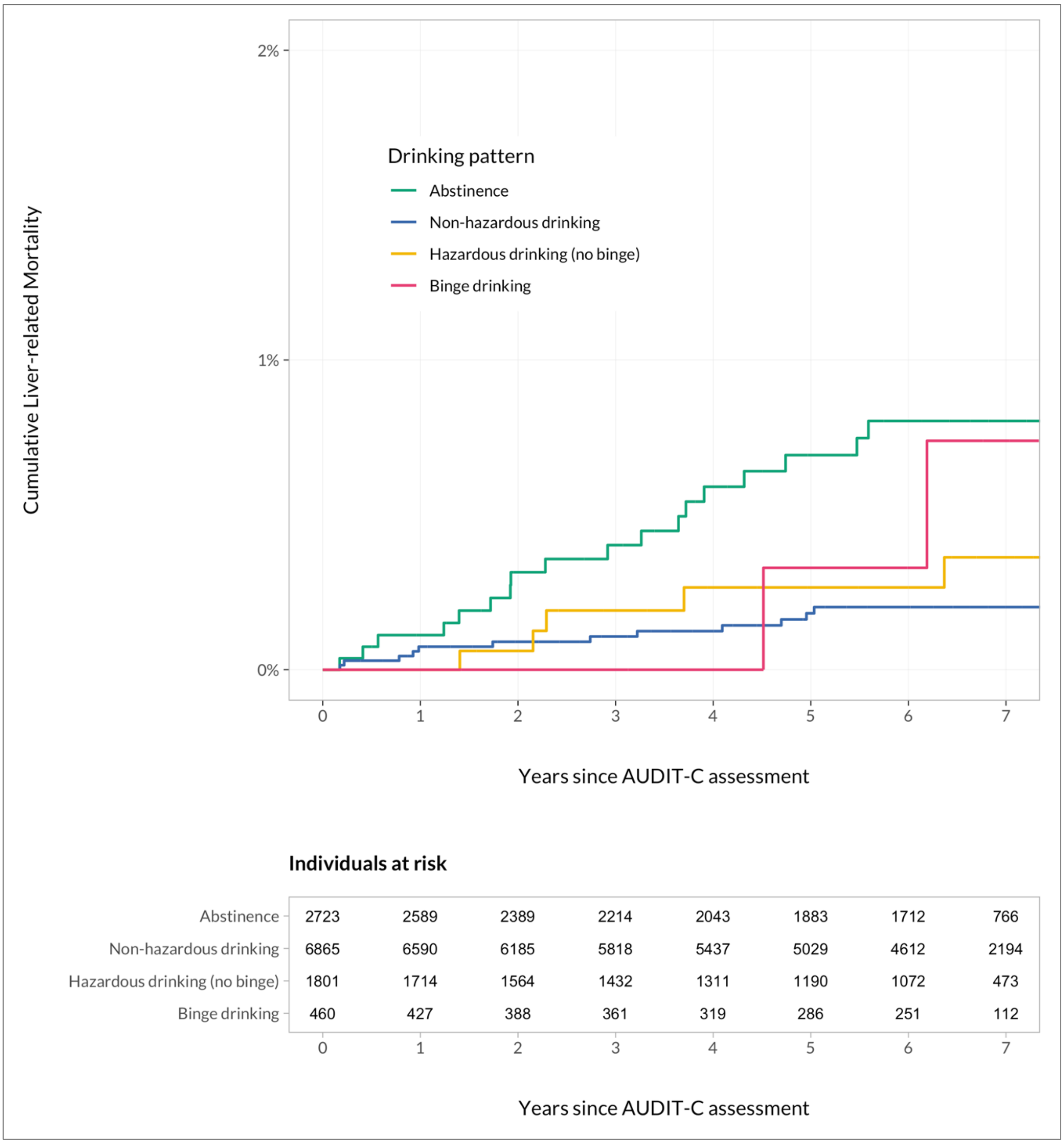

Figure S2. Kaplan Meier curves showing the time from the first AUDIT-C assessment to liver-related death, stratified by alcohol drinking pattern.

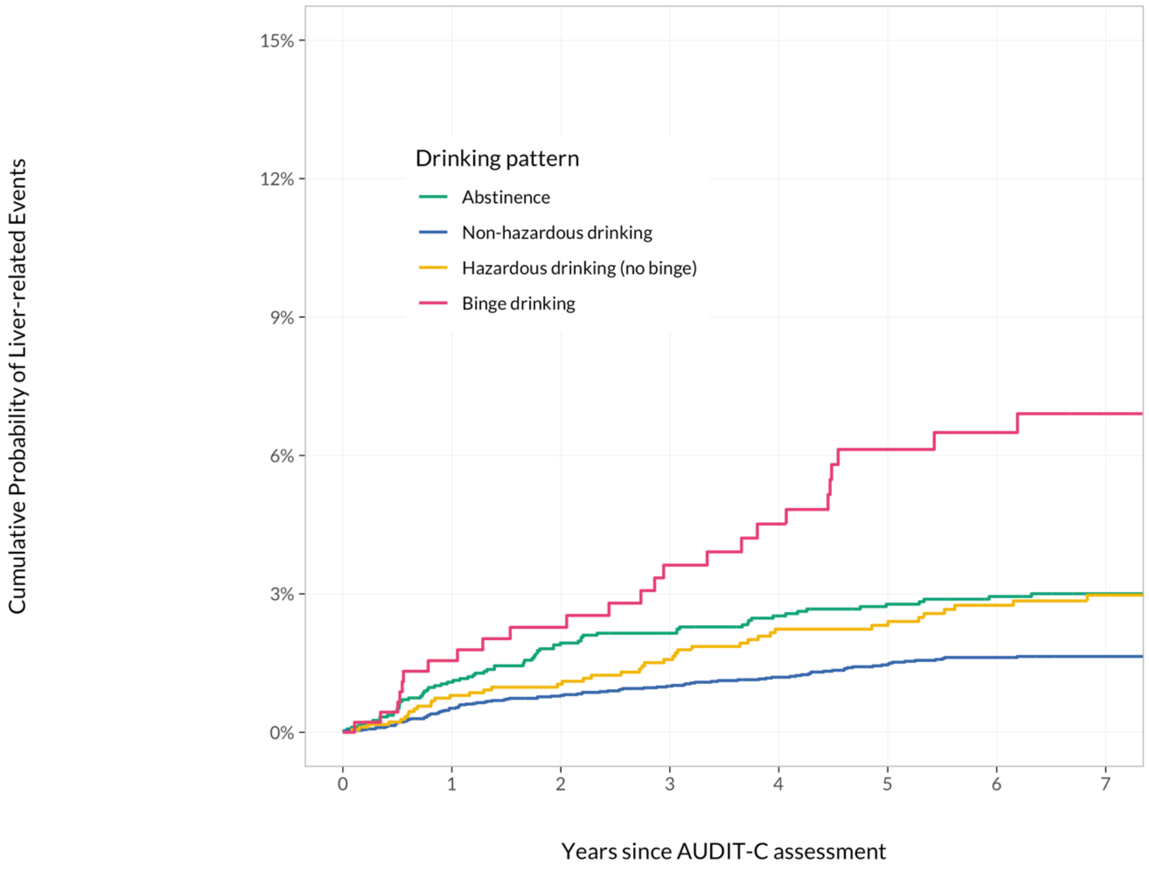

Individuals at risk

	0	1	2	3	4	5	6	7
Abstinence	2723	2561	2346	2169	1994	1836	1671	750
Non-hazardous drinking	6865	6558	6141	5765	5379	4964	4550	2162
Hazardous drinking (no binge)	1801	1700	1548	1411	1285	1167	1047	463
Binge drinking	460	420	380	349	306	271	237	108

Years since AUDIT-C assessment

Figure S3. Kaplan Meier curves showing the time from the first AUDIT-C assessment to the first liver-related event, stratified by alcohol drinking pattern.

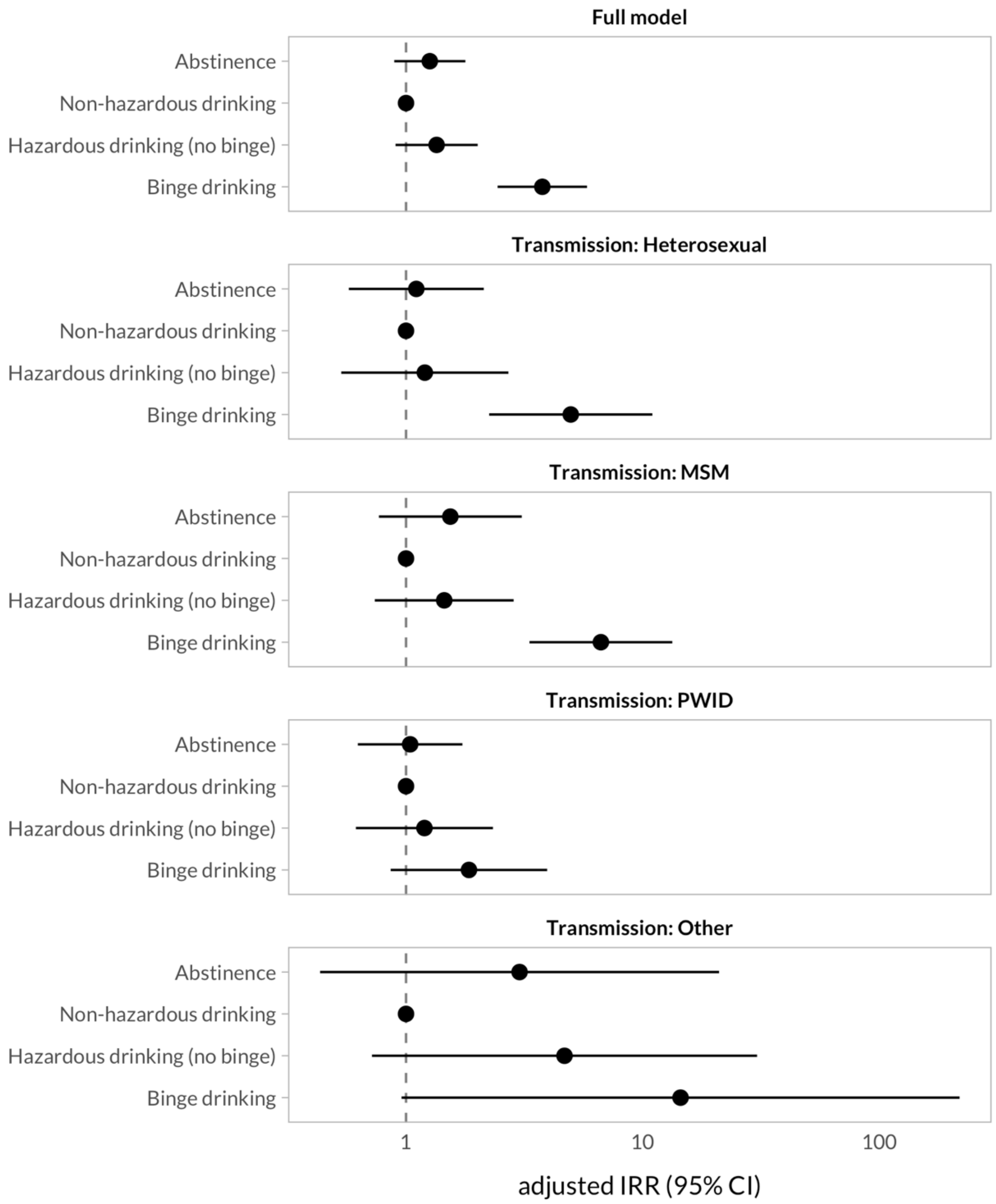

Figure S4. Subgroup analyses of the incidence rate ratios (**IRR**) for the occurrence of liver-related events by HIV transmission group. Whereas binge drinking was associated with the highest risk of liver-related events among heterosexual individuals and men who have sex with men (**MSM**), the impact was less pronounced among persons who inject drugs (**PWID**). Adjusted for age, sex, ethnicity, nadir CD4 cell count, education level, HBV and HCV coinfection, history of Aids defining disease, BMI, smoking, depression, employment status, partnership, and illicit drug use. **HBV** hepatitis B virus, **HCV** hepatitis C virus, **CDC** center for disease control.
